# Reforms in the Provision of Surgical Appliances

**Published:** 1913-05-24

**Authors:** 


### PROBLEMS IN ADMINISTRATIVE MEDICINE.

(Carefully thought-out Contributions to this Section will be welcome.)

### Reforms in the Provision of Surgical Appliances.

There are few of the many administrative problems, which hospital managers have to face, so varied and so troublesome as the supply of the many surgical and medical appliances ordered by the medical officers for in-patients or out-patients under their charge. As far as in-patients are concerned, the instruments required are, in a general hospital, chiefly splints of various kinds. These are kept in stock in large variety, are loaned to the patient during his treatment, and upon their return are cleaned, repadded by nurses, or renovated by the hospital carpenter. As a general rule no charge of any sort is made to the patient, however complicated and costly the apparatus may be, any more than for dressings, drugs, food, and the other necessaries of his case and his condition. At one time many surgeons desired all their patients to wear an abdominal belt after any operation upon the abdomen; and, as a rule, those patients whose means allowed it were expected to contribute part or the whole of the cost. But, nowadays, abdominal belts are much less extensively used than they were twelve or fifteen years ago; and this item does not bulk nearly so largely in hospital accounts.

The general principles on which in-patients are provided with surgical appliances in voluntary hospitals may be deduced, more or less roughly, from the two examples just given. That is, appliances which are merely loaned during a patient's stay are not charged for at all, even though they may be expensive, as, for instance, a Hodgen's splint apparatus; and those which the patient requires to take away with him when he goes out of the institution are charged for to such extent as is reasonable and practicable. It would be easy to quote plenty of exceptions, for the practice varies greatly at different hospitals; and there are many pieces of apparatus—plaster and silicate splints, for instance—which are made in the hospitals and taken away by the patient free of charge. Crutches, again, are often allowed to be taken away for short periods on loan, when the circumstances render it probable that the patient can be relied on to return them.

Appliances, in the main, can be arranged for in-patients without great difficulty in the majority of cases. They are ordered by the medical officer in charge of the case, who can easily see for himself whether fit, quality, and comfort have been attained. The patient can be instructed fully how to adjust and wear the particular appliance, and shown how to keep it clean and make it last well. The almoner can, without much risk of being imposed upon, judge accurately the financial circumstances of the patient or his family. And, in short, it is exceptional for complaints and dissatisfaction to arise. It should be added that at a great many hospitals Samaritan Funds exist for the benefit of those in-patients who really are destitute and in need of help on leaving the hospital, whether in the shape of clothes, food, railway fare, or surgical appliances.

In the out-patient departments a much less favourable side of the shield has to be exhibited. Here, apparatus of various kinds, especially orthopædic appliances, spectacles, and trusses, are prescribed by the out-patient surgeons. As a rule the patient has to take the prescription to some instrument maker approved by the hospital authorities, and usually there is supposed to be a very substantial reduction made off ordinary retail prices. It very often happens that this reduction is either illusory, or that it fails to bring the appliance in question within the means of the patient. Sometimes the Samaritan Fund of the hospital can give assistance; and, more often, the almoner is able to procure the necessary assistance. Indeed, this is one of the most important branches of an almoner's work, albeit a troublesome one. Some of the ways and means by which the almoner circumvents the difficulties will presently be discussed: for the moment we merely note that she is one of the most important links in the chain of supply. Let us suppose that somehow or other the financial problems connected with the acquisition of the necessary appliance have been overcome, and the order duly fulfilled by the instrument maker. The ideal institution is that in which the patient is then required to exhibit himself and his appliance to the surgeon who originally ordered it. Unfortunately, this is seldom carried out, for various reasons. Too often the patient, when instructed to this effect, neglects to present himself. When he does do so, not infrequently a cursory glance from some assistant house surgeon, who knows nothing of the case and very little about surgical apparatus, is all the inspection he gets. Sometimes, we regret to say, no effort is made to ensure that apparatus thus supplied shall be a reasonable fit and satisfactory for the purpose in view. Spectacles, especially for children, are liable to be carelessly fitted by hospital opticians when carrying out an out-patient order; and unless the ophthalmic surgeon makes a great point of seeing the patients after they have got their glasses, a great deal of bad work is done and much disappointment naturally follows its results.

Then, again, the average out-patient gives any surgical instrument committed to his keeping very heavy wear and tear. He may not make any attempt to keep it clean, or to make it last as long as possible by the "stitch in time," when it begins to show signs of wearing out. This is particularly the case with children. It is also noticeable that those patients who by reason of abject poverty have had instruments or appliances provided, free of cost to themselves, are much less likely to take care of them than are those who have had to pay part or the whole of the cost.

The result of all these unfavourable factors is that appliances for out-patients are apt to be unsatisfactory, either to the patient, the hospital, or the surgeon. A great deal can be done by the latter to remedy this state of affairs, though it means a certain amount of extra work and supervision on his part. The almoner's part in the transaction we have already referred to as all-important; for it is by her agency that the sinews of war have in many cases to be provided. "Letters" to surgical-aid funds and societies, sources of private charity which she knows how to tap for deserving patients, ways of interesting the clergy in their parishioners, and many other expedients can be made to serve her turn. In the Charity Organisation Society and its offspring, such as the Invalid Children's Aid Association, she has mainstays which seldom fail her in the last resort. The latter of these, usually abbreviated to "The I.C.A.A.," is a society which does an immense amount of excellent work, and one of its activities takes the form of providing surgical appliances for patients of tender years. Its organisation of local branches, worked by voluntary helpers, places the I.C.A.A. in an especially favourable position for the after-care of hospital cases. Thus, to give an example, a child with some serious deformity of the foot or leg is ordered by an out-patient surgeon to wear some particular kind of surgical boot. Not only does the I.C.A.A. in many cases pay part or the whole of the cost (recovering from the parents a small weekly sum if they are able to pay it), but it also watches the child's future, and sees to the mending of the boot at intervals as may be required. Instrument makers do not care for this sort of work, and they will undertake it as a rule only at high prices. The local branch of the I.C.A.A. often employs some respectable boot repairer in the district, who may be quite competent to resole a surgical boot even if he could not be entrusted with the original order for its construction; and much money is thus saved. Moreover, the visitor who calls at intervals to see how the cripple is getting on can do a lot towards making appliances of this sort last well by rebuking careless usage: they can also aid in the cure of the patient by reporting omissions to wear the particular splint or boot that has been ordered. Where this system has been most fully developed, the results of the use of surgical appliances ordered for children in out-patient departments are correspondingly the most encouraging, and the work is expanding year by year. Still, in the main, it is true that surgical appliances for out-patients are often difficult to obtain and do not effect the good they ought to.

One remedy is more co-ordination between the different departments concerned. Another, perhaps visionary, is the establishment of a joint hospital instrument maker's shop on co-operative lines.

